# Depression, anxiety and stress among flight crews in Tunisia

**DOI:** 10.1192/j.eurpsy.2024.1126

**Published:** 2024-08-27

**Authors:** Z. Athimni, I. Jammeli, A. Chouchane, I. Kacem, A. Aloui, M. Bouhoula, A. Gaddour, M. Maoua, A. Brahem, H. Kalboussi, O. El Maalel, S. Chatti

**Affiliations:** ^1^Occupational Medicine and Professional Pathologies Department, Farhat Hached Teaching Hospital, Avenue Ibn El Jazzar, Sousse, 4000, Tunisia, Sousse; ^2^Occupational Medicine Department- Ibn El Jazzar Hospital, Kairouan, Tunisia

## Abstract

**Introduction:**

The mental health of flight crews is of paramount importance. Due to the demanding nature of their work, crew members are subject to various stress factors such as irregular working hours, time differences, operational demands and high passenger safety responsibilities.

**Objectives:**

We aimed to evaluate the mental health of Tunisian flight crews working for a private airline.

**Methods:**

This is an exhaustive cross-sectional study which included all flight crews working for a private airline in Tunisia who consulted the occupational medicine and pathology department at the Farhad Hached University Hospital in Sousse as part of their periodic check-up. Data collection was based on a pre-established questionnaire which included socio-demographic data, lifestyle habits and professional data. The DASS21 questionnaire was used to assess depression, anxiety and stress.

**Results:**

Our study included 160 participants. The median age was 42 years with a female predominance. More than half were smokers (58.8%). Alcohol was consumed by 41.3% of flight crews. The vast majority drank coffee (84.4%). With regard to professional data, 71.3% were flight attendants. The median length of service was 15 years. The majority of participants had operated a medium-haul flight (< 5 hours) during the last month (65%). The majority of participants (85.6%) had a normal depression score. Almost a third of the participants (28.5%) had anxiety scores ranging from mild in 24 to extremely severe in one patient. The majority of flight crews had a normal stress score (90%). After multivariate analysis, unmarried marital status, working more than 2 days a week and stress were factors independently associated with anxiety.

**Conclusions:**

Work-related psychosocial risks can have a major impact on workers’ mental health. It is therefore essential to take these risks into account and put in place preventive measures to protect workers’ mental health and promote their well-being at work.

**Disclosure of Interest:**

None Declared

